# Postoperative Skeletal Stability and Pharyngeal Airway: Counterclockwise versus Clockwise Rotation during Mandibular Setback Surgery

**DOI:** 10.1155/2020/3283080

**Published:** 2020-01-30

**Authors:** Yu-Chuan Tseng, Szu-Yu Hsiao, Jung-Hsuan Cheng, Kun-Jung Hsu, Chun-Ming Chen

**Affiliations:** ^1^Graduate Institute of Dental Sciences, School of Dental Medicine, Kaohsiung Medical University, Kaohsiung, Taiwan; ^2^Department of Orthodontics, Kaohsiung Medical University Hospital, Kaohsiung, Taiwan; ^3^Department of Dentistry for Child and Special Needs, Kaohsiung Medical University Hospital, Kaohsiung, Taiwan; ^4^Department of Dentistry, Kaohsiung Municipal Ta-Tung Hospital, Kaohsiung, Taiwan; ^5^Department of Oral and Maxillofacial Surgery, Kaohsiung Medical University Hospital, Kaohsiung Medical University, Kaohsiung, Taiwan

## Abstract

**Purpose:**

To compare the effects of counterclockwise rotation (CCR) and clockwise rotation (CR) of the mandible on the pharyngeal airway during mandibular setback surgery. *Materials and Methods*. Serial cephalograms of 40 patients with mandibular prognathism, including 20 who underwent CCR and 20 who underwent CR, were taken at the following time intervals: preoperatively (T1), immediately postoperatively (T2), >1 year after surgery (T3), final surgical changes (T31), postoperative stability (T32), and immediate surgical change (T21). Changes in menton (Me) and hyoid (H) positions, soft palate width, soft palate length, soft palate angle and craniovertebral angle (C2C4-SN), and pharyngeal airway spaces (nasal pharyngeal airway (NOP), uvula pharyngeal airway (UOP), tongue pharyngeal airway (TOP), and epiglottis pharyngeal airway (EOP)) were evaluated.

**Results:**

The mean Me (T31) setback for CCR and CR was 12.56 and 13.06 mm, respectively, with 2.41 mm upward and 3.29 mm downward, respectively. The vertical Me position of CR exhibited significant downward movement compared with that of CCR. The mean H setback results for CCR and CR were 4.42 and 5.75 mm, respectively, with 1.47 mm downward and 2.97 mm downward, respectively. The C4C2-SN angles for CCR and CR increased by 2.68° and 3.65°, respectively, whereas their palatal angles increased by 2.35° and 5.25°, respectively. Pearson's correlation analysis (T31) revealed that for CCR, no pharyngeal airway spaces were significantly correlated with any measured variables. In CR, NOP was significantly correlated (*r* = 0.58) with the vertical Me position. Significant relapse (T32) was observed after CR in the horizontal (*r* = 0.58) with the vertical Me position. Significant relapse (T32) was observed after CR in the horizontal (*r* = 0.58) with the vertical Me position. Significant relapse (T32) was observed after CR in the horizontal (

**Conclusion:**

Pharyngeal airway space narrowed postoperatively, and its patency was appropriately maintained through natural physiological regulation of the craniovertebral angle (C2C4-SN). Significant postoperative relapse was correlated with CR.

## 1. Introduction

Regarding the relationships of pharyngeal airway space with various facial patterns, patients with mandibular prognathism have the most pharyngeal airway space, followed by healthy individuals and then patients with mandibular retrognathism [1]. In other words, a person's mandibular configuration affects the anteroposterior dimensions of their pharyngeal airway. According to anatomical studies [2, 3], postoperative changes in the pharyngeal airway occur between the soft palate and epiglottic cartilage. Several scholars [2–4] have reported that changes in tongue position are accompanied by those in mandibular position. Studies [4, 5] have shown that mandibular advancement surgery can shift the tongue forward and increase the size of the pharyngeal airway, whereas mandibular setback surgery narrows the pharyngeal airway. Thus, the tongue and its attached muscles may play crucial roles in maintaining the size of the pharyngeal airway. The soft palate, posterior border of the tongue, epiglottic cartilage, and cervical vertebrae are the primary influencing structures. Measuring changes in these structures could accurately reflect the clinical situation of an individual's postoperative pharyngeal airway.

Because of postoperative occlusal stability and aesthetic requirements, the mandible may be rotated in a counterclockwise rotation (CCR) or clockwise rotation (CR) according the occlusal plane. Yang and Hwang [6] investigated the correlation between postoperative relapse and intraoperative CR of the proximal segment after mandibular setback with sagittal split ramus osteotomy. Patients who had undergone high CR of the proximal segment demonstrated greater tendency for horizontal relapse than did patients who had undergone low CR of the proximal segment. Therefore, postoperative skeletal stability was affected by CCR and CR of the mandible and then influenced the postoperative pharyngeal airway space. The present study investigated the difference in postoperative pharyngeal airway space after mandibular setback surgery involving CCR and CR.

## 2. Materials and Methods

Forty patients with mandibular prognathism were recruited from the Oral and Maxillofacial Surgery Department of Kaohsiung Medical University Hospital (KMUH). The inclusion criteria were (1) no history of trauma or other congenital craniofacial abnormality, (2) no remaining active growth of the mandible, (3) mandibular setback (intraoral vertical ramus osteotomy) alone without maxillary or other surgery, and (4) maxillomandibular fixation for 6 weeks. The patients were divided into two groups: (1) 20 patients: CCR during mandibular setback surgery and (2) 20 patients: CR during mandibular setback surgery. The median age of CCR group (13 women and 7 men) and CR group (8 women and 12 men) was 22.5 and 20.5, respectively.

**Figure 1:**
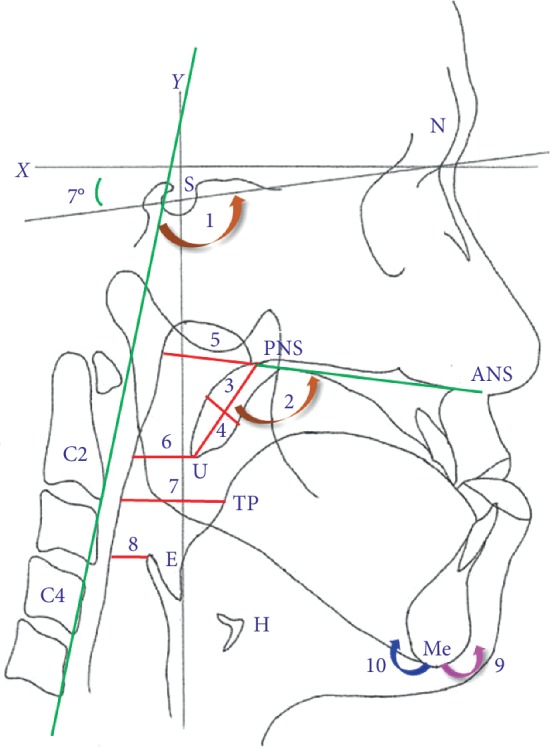
Landmarks, reference lines, distances, angles, and pharyngeal airway measurements. *X*, horizontal line; *Y*, vertical line; S, sella; N, nasion; Me, menton; H, hyoid bone; ANS, anterior nasal spine; PNS, posterior nasal spine; U, tip of uvula; TP, tongue posterior; C2, inferoanterior point on the second cervical vertebra; C4, inferoanterior point on the fourth cervical vertebra; E, most superior point on the epiglottis. Green lines: C2C4 line; ANS-PNS line. Brown arrow: (1) soft palate angle (ANS-PNS-U angle) and (2) cervical vertebral angle (C4C2-SN angle). Red lines: (3) SPL (length of the soft palate); (4) SPW (widest distance of the soft palate); (5) NOP (nasal pharyngeal airway); (6) UOP (uvula pharyngeal airway); (7) TOP (tongue pharyngeal airway); (8) EOP (epiglottis pharyngeal airway). Purple: (9) (counterclockwise rotation) Blue: 10 (clockwise rotation).

Serial cephalograms (T1: preoperation; T2: immediate postoperation; and T3: > 1 year postoperation) were obtained to examine pharyngeal airway depth, mandible, soft palate, cervical vertebrae, and tongue. The surgical changes were defined as follows: postsurgical immediate change (T21), final surgical changes (T31), and postoperative stability (T32). The reference points and definitions are shown in Figure 1. Landmarks were defined as follows: S, sella; N, nasion; Me, menton; H, hyoid bone; ANS, anterior nasal spine; PNS, posterior nasal spine; U, tip of the uvula; C2, inferoanterior point on the second cervical vertebra; C4, inferoanterior point on the fourth cervical vertebra; TP, tongue posterior (C2 horizontal line intersect with tongue posterior); and E, most superior point on the epiglottis. The three reference lines were as follows: (1) *X*-axis: constructed by drawing a line through N 7° above the SN line; (2) *Y*-axis: constructed by drawing a line through S perpendicular to the *X*-axis; and (3) C2C4 line. The following variables were measured: (1) nasal pharyngeal airway (NOP): ANS-PNS plane intersecting the pharyngeal wall; (2) uvula pharyngeal airway (UOP): distance between U and the pharyngeal wall; (3) tongue pharyngeal airway (TOP): distance between TP and the pharyngeal wall; (4) epiglottis pharyngeal airway (EOP): distance between E and the pharyngeal wall; (5) length of the soft palate (SPL): distance between U and the PNS; and (6) widest distance of the soft palate (SPW). The soft palate angle (ANS-PNS-U) and craniovertebral angle (angle between the C2C4 and SN lines) were also measured. The null hypothesis is that final postoperative (T31) pharyngeal airway space (NOP, UOP, TOP, and EOP) would not differ significantly between CCR and CR.

Postoperative changes at the reference points during each period (T21, T32, and T31) were quantified to estimate statistical parameters, including the mean and standard deviation. The directions of movement were defined in the horizontal (+: forward; −: backward) and vertical (+: downward; −: upward) directions. SPSS (version 20; IBM Corporation, Armonk, NY, USA) was employed for statistical analysis, and *p* < 0.05 was considered significant. Postoperative changes in landmarks in each period were identified for statistical analyses, involving means, standard deviations, and Student's *t*-test. Pearson's correlation coefficient analysis was performed to examine the statistical significance of correlations between changes in the pharyngeal airway space and those in the positions of the mandible, cervical vertebrae, and tongue. Regarding absolute correlation coefficient values, 0–0.19 was considered very weak, 0.2–0.39 was considered weak, 0.40–0.59 was considered moderate, 0.6–0.79 was considered strong, and 0.8–1 was considered very strong. This study was reviewed and approved by the Human Investigation Review Committee of KMUH (KMUH-IRB-20140173).

## 3. Results

The immediate postoperative changes (T21) of the CCR and CR groups are summarized in Table 1. The horizontal mandibular setback (Me) presented no significant difference between CCR (13.40 mm) and CR (12.62 mm). The vertical Me position for CR exhibited significant downward (3.87 mm) movement compared with that for CCR (upward: 0.06 mm). There is no significant difference between CCR and CR in the C4C2-SN angle, palatal angle, SPW, SPL, hyoid, and 4 pharyngeal airway spaces (NOP, UOP, TOP, and EOP). The postoperative stability (T32) of the CCR and CR groups is summarized in Table 2. There is no significant difference between CCR and CR in the all measurements.

The mean final postoperative changes (T31) of the CCR and CR groups are summarized in Table 3. The horizontal mandibular setback (Me) presented no significant difference between CCR (12.56 mm) and CR (13.06 mm). The vertical Me position for CR exhibited significant downward (3.29 mm) movement compared with that for CCR (upward 2.41 mm). The mean H setback for CCR and CR was 4.42 and 5.75 mm, respectively, with 1.47 and 2.97 mm downward, respectively. The C4C2-SN angles for CCR and CR increased by 2.68° and 3.65°, respectively. However, there is no significant difference between CCR and CR in the C4C2-SN angle, palatal angle, SPW, SPL, hyoid, and 4 pharyngeal airway spaces (NOP, UOP, TOP, and EOP). Therefore, the null hypothesis was accepted.

In Table 4, Pearson's correlation analysis (T31) revealed that no pharyngeal airway space measurements (NOP, UOP, TOP, and EOP) for CCR were significantly correlated with any of the measured variables (Me, H, SPW, SPL, C4C2-SN angle, and palatal angle). For CR, NOP was significantly moderately correlated (*r* = 0.58) with the vertical Me position; however, the other pharyngeal airway space measurements (UOP, TOP, and EOP) were not significantly correlated with any of the measured variables. In Table 5, horizontal relapse (Me-T32) of CR was significantly negative and strongly correlated (*r* = −0.72) with immediate postoperative setback (horizontal Me-T21). The vertical relapse (Me-T32) of CR was significantly negative and very strongly correlated (*r* = −0.82) with immediate postoperative setback (vertical Me-T21). The vertical relapse (Me-T32) of CCR was significantly negative and very strongly correlated (*r* = −0.91) with immediate postoperative setback (vertical Me-T21).

## 4. Discussion

The postoperative stability of CCR and CR during SSRO setback procedure was well documented. Yang and Hwang [6] reported that SSRO mandibular setback with high CR of the proximal segment demonstrated greater tendency for horizontal relapse. However, all studies were focused on the SSRO method and its proximal segment was fixed to distal segment using the miniplate and miniscrew. However, proximal segment of IVRO was not fixed to distal segment and it was passive contact with distal segment. It means that postoperative mandibular rotations (CCR and CR) were the distal segment in IVRO and the proximal segment in SSRO. Therefore, it was different mandibular segment (proximal or distal) rotations between IVRO and SSRO in the postoperative skeletal stability and pharyngeal airway space. Therefore, our study was the first report to present the outcomes of IVRO following CCR and CR.

Jena et al. [7] noted that the soft palate is significantly shorter in patients with mandibular prognathism than in those with mandibular retrognathism; in addition, the soft palate is significantly thicker in patients with mandibular prognathism than in individuals with normal mandibular development and patients with mandibular retrognathism. Turnbull and Battagel [8] and Muto et al. [9] reported that palate angle and SPL were increased after mandibular setback operation. Similar to previous report [8, 9], our study showed that both of CCR and CR had increased in the palate angle and SPL. This means that mandibular setback is associated with the traction of the palatal arch muscle; this leads to increased palate angle and elongate SPL. Moreover, our study found that neither CCR nor CR yielded significant changes in the soft palate dimensions (SPL, SPW, and palatal angle). Although no significant difference was observed between CCR and CR, the palatal angle for CR was greater than that for CCR immediately after surgery (T21) and on final measurement (T31).

When orthognathic surgery is performed to set back the mandible, the tongue, uvula, soft palate, and epiglottis shift backward postoperatively, leading to a narrowing of the pharyngeal airway. In particular, significant narrowing of the hypopharyngeal airway was evident on a lateral cephalogram. Hwang et al. [10] measured changes in the immediate postoperative airway at the level of the second cervical vertebra (setback: 3.2 mm) and observed narrowing of 0.67 mm. Eggensperger et al. [11] reported that immediate postoperative lower airway reduction (0.3 mm) had occurred at the level of the epiglottis (setback: 6.3 mm). Here, we found that the mandibular setback procedure narrowed the pharyngeal airway in the following order: TOP, UOP, EOP, and finally, NOP. Therefore, mandibular setback primarily narrowed the TOP and UOP.

Here, the clinical observations revealed that in an attempt to hide the appearance of mandibular protrusion, patients with mandibular protrusion tend to slightly lower their heads preoperatively compared with unaffected individuals. Regarding the relative postoperative changes in the position of a patient's head and cervical vertebrae, Marşan et al. [12] reported that patients with mandibular protrusion exhibited 6.3 mm setback after mandibular setback surgery, along with a significant increase of 3.7° in the craniovertebral angles 1 year postoperatively. In a postoperative 3-year follow-up study, Achilleos et al. [13] demonstrated that the craniovertebral angles significantly increased by 3.2°. In the present study, we found that the craniovertebral angle (C2C4-SN angle) had increased after both CR and CCR. The C2C4-SN angle exhibited an evident increase to counteract pharyngeal airway narrowing due to mandibular setback surgery. Thus, the head remained more erect compared with the preoperative position; this finding was consistent with the results of the aforementioned reports. Our results revealed that postoperative changes occurred in the relative positions of the head and cervical vertebrae in response to relevant structural and physiological changes after surgery. Although no significant differences were observed between CR and CCR outcomes, CR increased the C2C4-SN angle to a greater degree than did CCR. CR requires a wider postoperative C2C4-SN angle to compensate for the narrowing of the pharyngeal airway because upward movement of the distal ramus segment (CR) compresses the pharyngeal airway to a greater extent than does downward movement of distal ramus segment (CCR).

Pearson's correlation analyses were performed on final postoperative reductions (T31) in the NOP, UOP, TOP, and EOP lengths in the pharyngeal airway to examine their relationships with changes in the other immediate postoperative variables. In CCR, although Me exhibited significant setback, it had no significant correlations with NOP, UOP, TOP, or EOP. The results of CR were similar to those of CCR except that NOP was significantly positively correlated with the vertical change of Me. During mandibular setback surgery, the hyoid bone was accompanied first backward and then downward to counterbalance the compression of the pharyngeal airway. Although no significant differences between CR and CCR measurements were observed, greater pharyngeal airway compression was observed in CR, and more hyoid bone downward movement was found in CR than in CCR. However, the final postoperative changes in the hyoid bone (horizontal and vertical positions) had no significant correlations with changes in pharyngeal airway space for either CCR or CR; this finding indicated that the hyoid bone could adjust its position to balance the alternation of the pharyngeal airway.

The examination of the C4C2-SN angle revealed that CCR and CR had no significant correlations with pharyngeal airway space. After >1 year of postoperative follow-up, no correlations with reductions in the NOP, UOP, TOP, or EOP were observed. Therefore, postoperative pharyngeal airway space was unaffected by changes in the C4C2-SN angle. However, both CR and CCR evidently increased the C4C2-SN angle. This finding further indicated that pharyngeal airway space decreased postoperatively and pharyngeal airway patency was appropriately maintained through natural physiological regulation. Thus, maintenance of the pharyngeal airway after mandibular setback surgery is achieved through adaptive changes in head position, which could increase the dimension of the pharyngeal airway through an increment of craniocervical inclination [14–16]. Although postoperative soft palate-related structures (SPL, SPW, and soft palate angle) changed, they did not significantly influence pharyngeal airway space (NOP, UOP, TOP, or EOP) for either CCR or CR.

The examination of postoperative relapse (T32) revealed no differences between CR and CCR. However, Pearson's test revealed a highly significant negative correlation between the degree of mandibular setback (T21) and postoperative relapse (T32) in CR but not in CCR. Therefore, the rotation of mandibular setback demonstrated a greater impact on postoperative relapse than did the degree of mandibular setback; thus, CR compressed pterygomandibular sling more than CCR did. In summary, the mandibular setback procedure must consider the rotation of the mandible to minimize the risk of postoperative relapse.

In conclusion, the greater the degree of mandibular setback, the narrower is the pharyngeal airway. However, no significant correlation between the degree of mandibular setback and pharyngeal airway space was observed for either CCR or CR. We thus concluded that downward movement of the hyoid bone can offset the compression caused by mandibular setback. Moreover, the cervical vertebral angle significantly increases to compensate for the narrowed pharyngeal airway. Although the observed postoperative measurement changes were similar for CCR and CR, we observed a significant correlation between the degree of mandibular setback (T21) and postoperative relapse (T32) for CR.

## Figures and Tables

**Table 1:** The clockwise and counterclockwise rotation of mandibular setback in the immediate surgical change (T21) by Student's *t*-test.

Variables	Clockwise		Counterclockwise		Intergroup group comparison	
	Mean	SD	Mean	SD	*p* value	
C4C2-SN angle	4.93	4.60	3.18	1.08	0.42	—
Palatal angle	4.60	9.20	0.33	6.22	0.08	—
Soft palate width	0.27	3.33	0.17	1.96	0.87	—
Soft palate length	1.44	3.00	2.13	3.22	0.11	—
Pharyngeal airway						
NOP	−0.42	3.36	−1.04	1.73	0.48	—
UOP	−3.17	2.76	−2.11	2.69	0.25	—
TOP	−2.80	4.36	−2.77	3.82	0.98	—
EOP	−0.90	4.56	0.17	2.45	0.44	—
Hyoid						
Horizontal	−5.70	7.05	−4.05	6.58	0.46	—
Vertical	10.16	5.78	8.79	5.28	0.35	—
Menton						
Horizontal	−12.62	4.81	−13.40	3.70	0.58	—
Vertical	3.87	5.24	−0.06	4.69	0.04	^*∗*^

^*∗*^Statistically significant; *p* < 0.05; —: not significant.

**Table 2:** The clockwise and counterclockwise rotation of mandibular setback in the postoperative stability (T32) by Student's *t*-test.

Variables	Clockwise		Counterclockwise		Intergroup group comparison	
	Mean	SD	Mean	SD	*p* value	
C4C2-SN angle	−1.28	7.95	−0.38	4.09	0.61	—
Palatal angle	0.65	6.00	1.53	4.29	0.57	—
Soft palate width	−0.56	1.60	−0.38	1.99	0.76	—
Soft palate length	−2.01	3.30	−0.61	3.47	0.19	—
Pharyngeal airway						
NOP	1.32	3.10	0.86	2.34	0.62	—
UOP	0.35	2.60	−0.10	2.89	0.63	—
TOP	−0.89	4.96	0.15	3.10	0.54	—
EOP	−0.66	3.99	−0.94	2.38	0.80	—
Hyoid						
Horizontal	−0.05	6.09	−1.06	4.55	0.45	—
Vertical	−7.19	4.95	−6.55	5.37	0.71	—
Menton						
Horizontal	−0.44	3.50	0.40	3.83	0.54	—
Vertical	−0.58	4.77	−1.74	4.69	0.47	—

^*∗*^Statistically significant; *p* < 0.05; —: not significant.

**Table 3:** The clockwise and counterclockwise rotation of mandibular setback in the final surgical changes (T31) by Student's *t*-test.

Variables	Clockwise		Counterclockwise		Intergroup group comparison	
	Mean	SD	Mean	SD	*p* value	
C4C2-SN angle	3.65	5.35	2.68	5.44	0.58	—
Palatal angle	5.25	6.58	2.35	5.97	0.13	—
Soft palate width	−0.29	1.71	−0.43	1.65	0.82	—
Soft palate length	1.31	2.70	0.99	4.01	0.76	—
Pharyngeal airway						
NOP	−0.16	1.95	−0.10	2.08	0.20	—
UOP	−2.26	2.06	−2.83	2.36	0.37	—
TOP	−3.11	3.06	−3.69	4.31	0.64	—
EOP	−1.51	3.85	−0.87	2.55	0.50	—
Hyoid						
Horizontal	−5.75	4.06	−4.42	6.12	0.47	—
Vertical	2.97	6.38	1.47	5.98	0.36	—
Menton						
Horizontal	−13.06	3.32	−12.56	4.29	0.63	—
Vertical	3.29	3.01	−2.41	1.66	<0.01	^*∗*^

^*∗*^Statistically significant; *p* < 0.05; —: not significant.

**Table 4:** Pearson test of clockwise and counterclockwise rotation of mandibular setback in the final surgical changes (T31).

	Clockwise				Counterclockwise			
	NOP	UOP	TOP	EOP	NOP	UOP	TOP	EOP
C4C2-SN angle	−0.23	0.42	0.36	0.18	0.30	−0.01	0.38	0.00
Palatal angle	−0.17	−0.24	−0.41	−0.18	−0.08	−0.27	−0.35	−0.07
Soft palate width	−0.22	0.04	−0.02	−0.12	0.32	0.09	0.20	−0.09
Soft palate length	0.03	−0.03	−0.33	−0.03	−0.28	0.23	−0.32	0.25
Hyoid								
Horizontal	0.16	−0.39	−0.14	0.00	0.04	0.01	−0.18	0.17
Vertical	−0.12	0.08	−0.03	0.36	0.01	−0.11	0.10	0.12
Menton								
Horizontal	0.41	0.08	0.29	0.08	0.41	−0.03	0.12	0.22
Vertical	0.58^*∗*^	0.05	0.33	0.13	−0.18	0.02	−0.01	−0.08

^*∗*^Statistically significant; *p* < 0.05; —: not significant.

**Table 5:** Pearson test of clockwise and counterclockwise rotation in the relapse (horizontal Me-T32 and vertical Me-T32).

Variable	Horizontal Me-T32		Vertical Me-T32	
	*r*	*p*	*r*	*p*
Clockwise rotation				
Horizontal Me-T21 (mm)	−0.72	<0.01^*∗*^	0.08	0.74
Vertical Me-T21 (mm)	0.19	0.42	−0.82	<0.01^*∗*^
Counterclockwise rotation				
Horizontal Me-T21 (mm)	−0.41	0.07	0.10	0.68
Vertical Me-T21 (mm)	−0.23	0.34	−0.91	<0.01^*∗*^

^*∗*^Statistically significant; *p* < 0.05; T21: immediate surgical changes; T32: final postoperation stability.

## Data Availability

The data used to support the findings of this study are included within the article. The data used to support the findings of this study are available from the corresponding author upon request.

## References

[1] Muto T., Yamazaki A., Takeda S. (2008). A cephalometric evaluation of the pharyngeal airway space in patients with mandibular retrognathia and prognathia, and normal subjects. *International Journal of Oral and Maxillofacial Surgery*.

[2] Kawamata A., Fujishita M., Ariji Y., Ariji E. (2000). Three-dimensional computed tomographic evaluation of morphologic airway changes after mandibular setback osteotomy for prognathism. *Oral Surgery, Oral Medicine, Oral Pathology, Oral Radiology, and Endodontology*.

[3] Greco J. M., Frohberg U., Van Sickels J. E. (1990). Long-term airway space changes after mandibular setback using bilateral sagittal split osteotomy. *International Journal of Oral and Maxillofacial Surgery*.

[4] Enacar A., Aksoy A. U., Sencift Y., Haydar B., Aras K. (1994). Changes in hypopharygeal airway space and in tongue and hyoid bone positions following the surgical correction of mandibular prognathism. *The International Journal of Adult Orthodontics & Orthognathic Surgery*.

[5] Riley R. W., Powell N. B., Guilleminault C. (1990). Maxillary, mandibular, and hyoid advancement for treatment of obstructive sleep apnea: a review of 40 patients. *Journal of Oral and Maxillofacial Surgery*.

[6] Yang H. J., Hwang S. J. (2014). Contributing factors to intraoperative clockwise rotation of the proximal segment as a relapse factor after mandibular setback with sagittal split ramus osteotomy. *Journal of Cranio-Maxillofacial Surgery*.

[7] Jena A. K., Singh S. P., Utreja A. K. (2010). Sagittal mandibular development effects on the dimensions of the awake pharyngeal airway passage. *The Angle Orthodontist*.

[8] Turnbull N. R., Battagel J. M. (2000). The effects of orthognathic surgery on pharyngeal airway dimensions and quality of sleep. *Journal of Orthodontics*.

[9] Muto T., Yamazaki A., Takeda S., Sato Y. (2008). Effect of bilateral sagittal split ramus osteotomy setback on the soft palate and pharyngeal airway space. *International Journal of Oral and Maxillofacial Surgery*.

[10] Hwang S., Chung C. J., Choi Y.-J., Huh J.-K., Kim K.-H. (2010). Changes of hyoid, tongue and pharyngeal airway after mandibular setback surgery by intraoral vertical ramus osteotomy. *The Angle Orthodontist*.

[11] Eggensperger N., Smolka W., Iizuka T. (2005). Long-term changes of hyoid bone position and pharyngeal airway size following mandibular setback by sagittal split ramus osteotomy. *Journal of Cranio-Maxillofacial Surgery*.

[12] Marşan G., Öztaş E., Cura N., Kuvat S. V., Emekli U. (2010). Changes in head posture and hyoid bone position in Turkish class III patients after mandibular setback surgery. *Journal of Cranio-Maxillofacial Surgery*.

[13] Achilleos S., Krogstad O., Lyberg T. (2000). Surgical mandibular setback and changes in uvuloglossopharyngeal morphology and head posture: a short- and long-term cephalometric study in males. *The European Journal of Orthodontics*.

[14] Muto T., Takeda S., Kanazawa M., Yamazaki A., Fujiwara Y., Mizoguchi I. (2002). The effect of head posture on the pharyngeal airway space (PAS). *International Journal of Oral and Maxillofacial Surgery*.

[15] Winnberg A., Pancherz H., Westesson P.-L. (1988). Head posture and hyo-mandibular function in man. *American Journal of Orthodontics and Dentofacial Orthopedics*.

[16] Chen C. M., Lai S., Chen K. K., Lee H. E. (2015). Correlation between the pharyngeal airway space and head posture after surgery for mandibular prognathism. *BioMed Research International*.

